# Effects of Salinity on the Reproductive and Lifespan Traits of *Artemia* Parthenogenetic Lineages with Different Ploidy Levels

**DOI:** 10.3390/biology14081055

**Published:** 2025-08-15

**Authors:** Alireza Asem, Yuxin Li, Xintong Yan, Yaojia Zhang, Yunlong Zhu, Behrooz Atashbar Kangarloei, Chaojie Yang

**Affiliations:** 1 Yazhou Bay Innovation Institute, Hainan Tropical Ocean University, Sanya 572000, China; asem.alireza@gmail.com (A.A.); yuqixinyu@163.com (Y.L.); 122126898114@163.com (X.Y.); zyj15907989363@163.com (Y.Z.); z2949335394@163.com (Y.Z.); 2 Department of Ecology and Aquatic Stocks Management, *Artemia* and Aquaculture Research Institute, Urmia University, Urmia 57179-44514, Iran

**Keywords:** brine shrimp, life history, asexual, oviparity, ovoviviparity

## Abstract

Although previous studies have investigated the reproductive (performance and mode) and lifespan traits of parthenogenetic *Artemia*, ploidy level has not been considered. Four parthenogenetic *Artemia* lineages, i.e., diploid, triploid, tetraploid, and pentaploid, were examined to determine the role of ploidy level under osmotic stress conditions (50, 100, and 150 ppt). Although the reproductive mode of the pentaploid lineage is unaffected by changes in salinity, it is entirely switched to oviparity in the diploid lineage at 50 ppt and in the tetraploid lineage at 100 ppt. Moreover, tetraploid reproduction is completely inhibited at 50 ppt. Although oviparity has been proposed as an adaptive strategy enhancing *Artemia* fitness at high salinities, the exclusive oviparous reproduction observed in the diploid parthenogenetic lineage at 50 ppt suggests that low salinity may also act as an environmental stressor, driving oviparity to ensure the next generation. On the basis of lifespan data, the tetraploid lineage presents greater euryhalinity than other ploidy levels do, whereas the pentaploid lineage is more stenohaline. Additionally, discriminant function analysis revealed that diploid and tetraploid lineages display heterogeneous reproductive/lifespan patterns across salinities, whereas triploid and pentaploid lineages exhibit homogeneous patterns within their respective groups. Our findings challenge the prevailing view that the ploidy level of parthenogenetic *Artemia* is correlated with tolerance to critical hypersaline conditions. In conclusion, we propose that the life history of parthenogenetic *Artemia* is influenced not only by ploidy level but also by the multifactorial integration of environmental conditions (particularly salinity and temperature) and local intra-variation/adaptation within isolated habitats.

## Simple Summary

This study examined the responses of four parthenogenetic *Artemia* lineages (diploid, triploid, tetraploid, and pentaploid) to varying salinity levels, with a focus on reproductive traits and lifespan. Lifespan data revealed that the tetraploid lineage demonstrated a greater ability to tolerate a wide range of salinities, whereas the pentaploid lineage tolerated only a narrow range of salinity. The diploid and tetraploid lineages presented heterogeneous reproductive/lifespan traits across salinities, whereas the triploid and pentaploid lineages presented homogeneous responses within their groups. Our results do not support a clear correlation between ploidy and reproductive/lifespan traits.

## 1. Introduction

The genus *Artemia* Leach, 1819 inhabits saline and hypersaline environments and has a wide distribution on five continents. It comprises nine species and obligatory parthenogenetic lineages with four different ploidy levels (di-, tri-, tetra- and pentaploid) [1,2]. The parthenogenetic lineages of *Artemia* are maternally polyphyletic groups [2,3,4,5] that are distributed in the Old World and Oceania [6,7,8,9,10,11,12]. Although asexual *Artemia* are commonly referred to with the binomen “*Artemia parthenogenetica*”, they do not fit Mayr’s “Biological Species Concept” [2]. Asem et al. [2] suggested the use of the term “parthenogenetic lineage(s)” to describe asexual forms of *Artemia*.

Owing to the nutritional importance of *Artemia* as a live food, it has been extensively used in the aquaculture industry and in fish hatcheries [13,14,15]. Since the 20th century, American *Artemia franciscana* from Great Salt Lake (Utah, USA) and San Francisco Bay (California, USA) have been intentionally introduced into nonindigenous habitats [13]. While exotic *A. franciscana* is known as an economic species with high reproductive capacity and superior adaptability (see [16,17]), previous studies have demonstrated that the reproductive performance and fitness of native Egyptian and Iranian parthenogenetic *Artemia* are greater than those of *A. franciscana* in nonnative habitats [12,18].

Since the 1980s, global experimental efforts have sought to characterize the reproductive potential of *Artemia* species (including *A. salina*, *A. urmiana*, *A. franciscana*, *A. persimilis*, and *A. sinica*) and parthenogenetic lineages (e.g., [19,20,21,22,23,24,25,26,27,28,29,30,31,32]), but ploidy levels have received little attention. Early research on reproductive patterns and modes in *Artemia* dates back to Browne [33] in 1980, which focused on American *A. franciscana* and parthenogenetic *Artemia* from two localities in India (Madras and Kutch). Although Barigozzi [34] had identified different ploidy levels in obligate parthenogenetic lineages in 1974, this aspect was not considered in Browne’s 1980 study [33]. Browne et al. [19] expanded the study of reproductive characteristics in *Artemia* by including two European (Cádiz, Spain, and Giraud, France) and one Asian parthenogenetic *Artemia* (Izmir, Turkey). While the *Artemia* lineage from Giraud had previously been identified as diploid, the ploidy levels of the others remained unknown. The coexistence of parthenogenetic lineages with different ploidy levels within the same habitat should thus be considered a hidden problem causing confusion in the results. This issue is further complicated by the fact that parthenogenetic *Artemia* are not evolutionarily monophyletic (see [2,3,4,5]). For example, the parthenogenetic *Artemia* from the Tanggu salterns, China, examined by Triantaphyllidis et al. [21], consisted of a mixture of diploid, tetraploid, and pentaploid lineages. This problem is also evident in studies on parthenogenetic *Artemia* from Urmia Lake and AiBi Lake, in which different ploidy levels have been reported from the same localities (see [8,12]). Ubaskin et al. [35] examined the reproductive performance of parthenogenetic *Artemia* from nine salt lakes in Kazakhstan. The results revealed significant variation among females (in terms of the number of eggs in the brood pouch) in single-site samples from each lake. However, owing to a lack of information on ploidy levels, it is unclear whether the observed differences in each lake resulted from the combination of parthenogenetic lineages with different ploidy levels or from individual properties affected by within-group competition. Except for a study on a clonal tetraploid lineage by Abatzopoulos et al. [25], there is no comprehensive investigation of standard reproductive characteristics in parthenogenetic *Artemia* with confirmed ploidy levels. Therefore, there is a gap in the understanding of the role of ploidy level in the reproductive strategies of parthenogenetic *Artemia* compared to bisexual *Artemia* species.

During a pretest in which diploid parthenogenetic lineages of *Artemia* from Ga Hai Lake (China) were cultured, we observed unexpected results at 28 °C; at low salinity, the reproductive mode of the females fully changed to oviparity (see Results and Discussion). Salinity, temperature, and interaction effects are the primary abiotic ecological factors influencing the biology of *Artemia* [24,25,28,32,36]. The scarcity of hatchable wild eggs from other ploidy levels prevented us from investigating how salinity and temperature interact in this system. Therefore, the present study focused on a preliminary assessment of the reproductive potential and lifespan traits of parthenogenetic lineages of different ploidy levels with the temperature held constant at 28 °C.

To address this objective, parthenogenetic lineages from two inland salt lakes (Ga Hai Lake and Hoh Lake) and two salterns (Ankiembe and Yinggehai) were examined.

Inland Ga Hai Lake is a semiarid terminal salt lake located in Qinghai Province (China) with an altitude of 2870 m [37,38]. Its surface area ranged from 28.37 to 38.57 km^2^ from 1975 to 2020 [38]. The lake hosts a diploid parthenogenetic lineage [4,37,39,40].

Inland Hoh Lake is located in Qinghai Province (China) and has an altitude of 2995 m [37] and a surface area of approximately 120 km^2^ [41]. The lake hosts a tetraploid parthenogenetic lineage [4,42].

The coastal Ankiembe saltern is located 5 km south of Toliara city in Madagascar [43] and has an area of approximately 2 km^2^ [41]. The parthenogenetic lineage inhabiting this site was determined to be triploid [43].

The Yinggehai saltern is a marine solar saltern near Sanya city in Hainan Province (China) with an area of 36 km^2^ [41]. In a previous study, pentaploid (97.3%) and tetraploid (2.7%) parthenogenetic lineages were reported at this saltern [44]. Moreover, in the present study, we found a single triploid individual (see Materials and Methods).

This preliminary study investigates how *Artemia* parthenogenetic lineages of differing ploidy levels respond to varying salinity levels by focusing on reproductive (mode and output) and lifespan traits and their interdependencies. This study aimed to clarify the role of ploidy in shaping reproductive potential and life-history strategies under osmotic stress.

## 2. Materials and Methods

Four parthenogenetic lineages of *Artemia* with different ploidy levels were investigated. Further information is summarized in Table 1. Given the low reported frequency (2.7%) of the tetraploid lineage in the Yinggehai saltern, the ploidy levels of offspring from the 35 individuals examined from each treatment were assessed using karyotype analysis. To determine the ploidy level, the C-banding method was applied following the protocol of Yarmohammadi & Pourkazemi [45]. All the prepared slides were examined under a Nikon Eclipse 600 microscope, and chromosome counts were performed to assess ploidy levels. Only one triploid individual, which was removed, was found at 150 ppt.

The wild eggs were hatched in 0.4 µm filtered South China Sea water (salinity 33–34 ppt) under optimal conditions [46]. Newly hatched nauplii were cultured according to a previously described standardized laboratory method [47]. Given that previous studies on reproductive and lifespan traits have not established standardized methods for salinity selection, we chose our experimental levels on the basis of practical constraints related to the availability of wild samples (see Introduction) and aimed to cover the main salinity range reported in the literature. Additionally, we included both low- and high-salinity extremes that still permit *Artemia* to complete its life cycle and reproduce successfully. Accordingly, we selected 50 ppt as the lower limit and 150 ppt as the upper limit. To ensure a consistent pattern of salinity variation, we also included a midpoint value of 100 ppt. Thus, three salinity treatments (50 ppt, 100 ppt, and 150 ppt) were used in this experiment. To prepare salinities, sea salt was added to seawater from the South China Sea (Sanya coast: 18°15′ N 109°30′ E), and a handheld optical refractometer was used to ensure that the salinity reached the intended levels in each treatment (Extech Co., Shanghai, China). The photoperiod and temperature were 12 h L/12 h D and 28 °C, respectively. A mixed diet of *Dunaliella salina* and LANSY ZM (INVE, Phichit, Thailand) was supplied in accordance with a previously described feeding protocol [47].

Each premature female was cultured in a 50 mL transparent plastic Falcon tube upon the appearance of visible spots along its ovary (see [48]). Thirty-five females from each ploidy lineage were examined at each salinity (several individuals in some treatments died prior to reaching maturity; see Table 2). In total, ten reproductive and lifespan traits were recorded, including the pre-reproductive period, reproductive period, post-reproductive period, number of broods per female, interval between broods, number of nauplii, number of eggs, total offspring, number of offspring per brood, and lifespan.

While previous studies (e.g., [25]) classified the pre-reproductive, reproductive, and post-reproductive periods along with lifespan as life history traits, we treated the first three phases as reproductive traits. This distinction is based on the definition of “lifespan” (the length of time an organism lives or an object functions) and the fact that the first three phases are not influenced by lifespan (see Discussion). Furthermore, the oviparity percentage (the percentage of encysted embryos for each female, see [25,32]) was separately considered the “reproductive mode” distinct from the other nine reproductive traits because non-significant statistical outcomes may mask significant differences in the “number of eggs per female” across treatments, and vice versa. Such overlap could lead to false convergence in the discriminant analysis.

Following the “central limit theorem” concept, the results were analyzed within lineages (among different salinities for each ploidy) and between lineages (across different ploidy levels at the same salinity) by ANOVA (Tukey’s test, *p* < 0.05). Discriminant analysis was performed to evaluate within-lineage, between-lineage, and mixed-group (among ploidies and salinities) differentiation. The computer program SPSS 28 (https://www.ibm.com/support/pages/downloading-ibm-spss-statistics-28010; accessed on 17 June 2025) was used for statistical analysis.

## 3. Results

### 3.1. Within-Lineage

The results of ten reproductive and lifespan traits from four parthenogenetic lineages with different ploidy levels cultured at different salinities are summarized in Table 2 (see also Table S1). Comparisons of the studied traits among different salinities revealed highly significant differences among the different salinities (within-lineage) in the diploid and tetraploid lineages. Except for the post-reproductive period, the number of nauplii in the diploid lineage, the number of nauplii, and the lifespan in the tetraploid lineage, eight traits significantly differed among all three studied salinities at both ploidy levels (*p* < 0.05). Statistical analyses across different salinity levels revealed that five and three of the ten traits examined were not significantly different in the triploid lineage (i.e., post-reproductive period, interval between broods, number of nauplii, total offspring and offspring per brood) and in the pentaploid lineage (i.e., post-reproductive period, interval between broods and number of eggs), respectively (*p* > 0.05). Unexpected results were observed in the diploid lineage at 50 ppt and in the tetraploid lineage at salinities of 50 ppt and 100 ppt, in which females did not produce nauplii. The average lifespans of the individuals in these treatments were 58.26, 89.80, and 82.66, respectively. Moreover, the tetraploid lineage did not produce eggs at 50 ppt salinity. Except for the pre-reproductive period (49.16) and the number of nauplii (14.35) in the diploid and pre-reproductive periods in the tetraploid (88.80) and pentaploid (24.11) lineages, the studied traits generally presented low, non-significant values at 150 ppt in the diploid lineage and 50 ppt in the other lineages.

**Figure 1:**
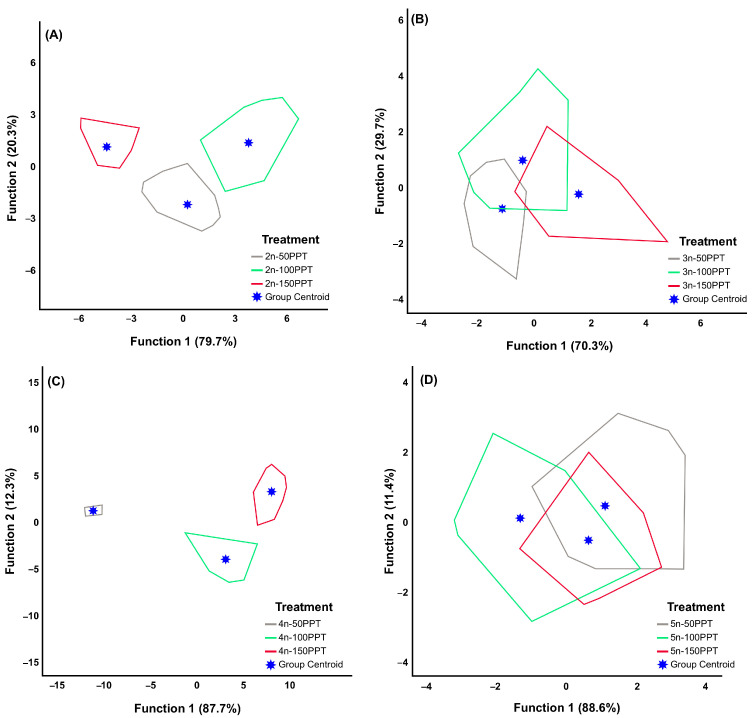
Scatterplot of the first two canonical discriminant functions resulting from within groups (with different salinities at the same ploidy level) from the discriminant analysis. (**A**) Diploid, (**B**) triploid, (**C**) tetraploid, and (**D**) pentaploid (the individuals corresponding to each polygon are presented in Figure S1).

The scatterplots of the discriminant analysis for each parthenogenetic lineage at different salinities are shown in Figure 1. The pentaploid (88.6%) and triploid (70.3%) lineages presented the highest and lowest percentages of variation in the first function, respectively. The two functions accounted for 100% of the variation at all ploidy levels. In the first discriminant function, the mean values of the reproductive period in diploid (1.386), the number of broods per female in triploid (1.563) and tetraploid (2.601), and the pre-reproductive period in pentaploid (0.812) were the primary drivers of group classification (Table S2). As shown in Figure 1, three distinct groups emerged within the diploid and tetraploid lineages. While 97.1% of the diploid individuals were correctly classified at 100 ppt, 100% of the diploid and tetraploid individuals in the other salinity treatments were correctly classified within their respective groups. The pentaploid lineage represented the lowest percentage (57.1%) of correctly classified individuals at 50 ppt. The tetraploid lineage presented the highest overall group classification accuracy (100%), and the pentaploid lineage presented the lowest classification accuracy (68.1%) (Table 3).

**Figure 2:**
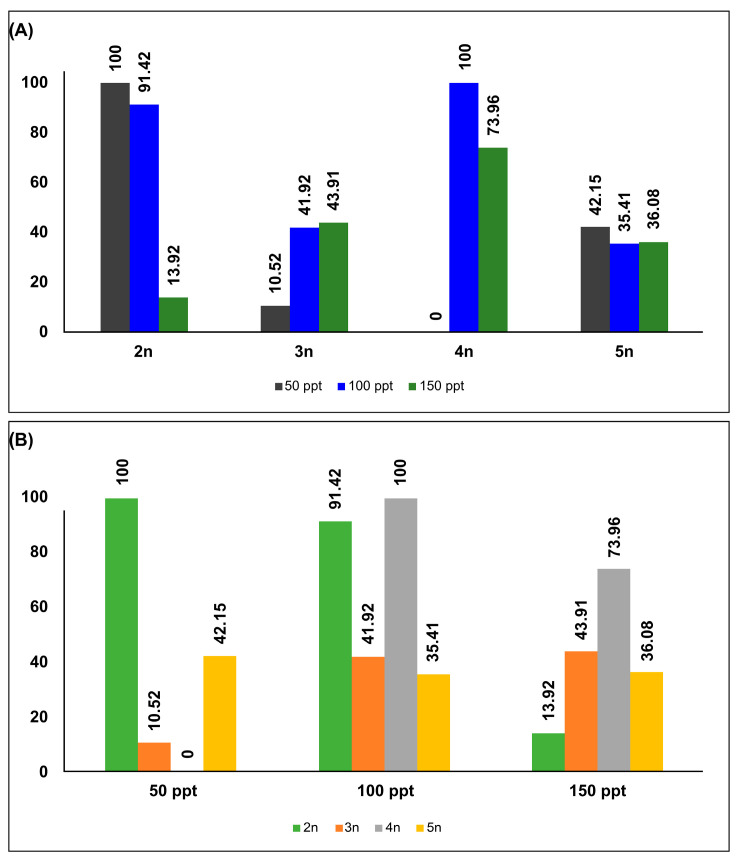
Percentages of oviparous female offspring: (**A**) at different salinities and at the same ploidy level (within-lineage) and (**B**) among different ploidy levels at the same salinity (between-lineage).

Findings on the reproductive mode (percentages of oviparity) are presented in Figure 2A and Table S3. These findings indicate that there is no correlation between salinity and reproductive mode within ploidy levels. While the diploid lineage displayed exclusively oviparous reproduction (100%) at the lowest salinity level (50 ppt), it increasingly shifted toward ovoviviparity under high-salinity conditions (150 ppt). In the triploid lineage, the maximum percentages of oviparous eggs were recorded at 150 ppt (43.91%) and 100 ppt (41.92%). Low salinity (50 ppt) thoroughly inhibited reproduction in the tetraploid lineage, whereas medium salinity (100 ppt) resulted in fully oviparous reproduction (100%). At high salinity (150 ppt), there was a tendency toward oviparity (73.96%) in the tetraploid. In contrast, all the treatments of the pentaploid lineage tended toward both oviparity and ovoviviparity, with no significant differences observed across salinities.

### 3.2. Between-Lineages

Table 4 presents the statistical analyses of reproductive and lifespan traits for the three salinities at different ploidy levels. At 150 ppt, five of the ten measured traits (i.e., reproductive period, number of broods per female, total offspring, offspring per brood and lifespan) presented significant differences (*p* < 0.05) across parthenogenetic lineages; the highest values were observed in the tetraploid lineage, whereas the lowest values were found in the diploid lineage, except for the lifespan, which was shortest in the pentaploid lineage (Table 2 and Table 4). There was no correlation between salinity and ploidy level, and high and low values for reproductive traits were distributed among different ploidy levels at each salinity. Notably, the highest values of reproductive traits were recorded in the tetraploid lineage at different salinities, including the pre-reproductive period at 50 ppt (88.80), the post-reproductive period at 100 ppt (21.51), the reproductive period (65.23), the number of broods per female (31.17), the number of eggs (232.97), the total number of offspring (315.77) and the number of offspring per brood (24.14) at 150 ppt.

**Figure 3:**
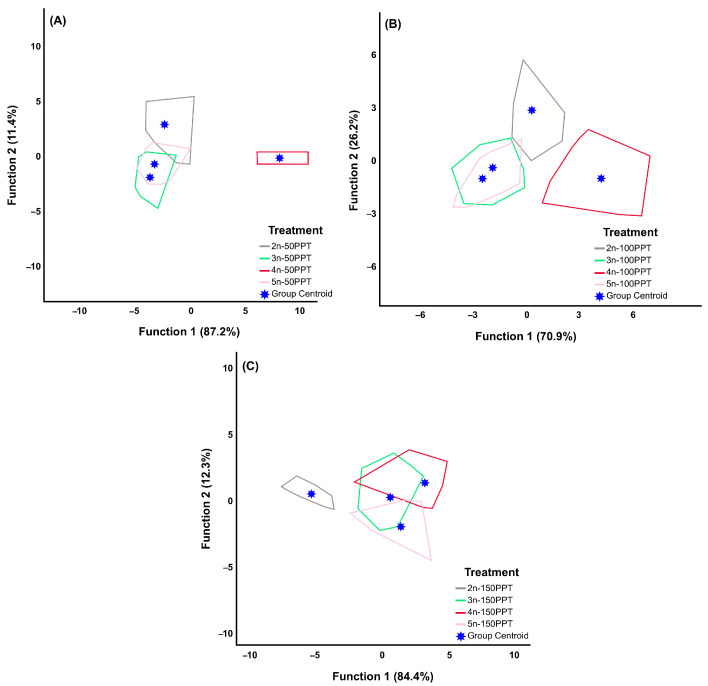
Scatterplot of the first two canonical discriminant functions resulting from among groups (among ploidy levels at the same salinity) from the discriminant analysis. (**A**) 50 ppt, (**B**) 100 ppt, and (**C**) 150 ppt (the individuals corresponding to each polygon are presented in Figure S2).

Figure 3 displays the scatterplots from the discriminant analysis comparing different parthenogenetic lineages under the same salinity conditions. The results indicate that the first discriminant function accounted for the highest percentage of variation at 50 ppt salinity (87.2%) and the lowest at 100 ppt salinity (70.9%). Furthermore, the cumulative variation explained by the first and second discriminant functions reached 98.6% at 50 ppt and 97.1% at 100 ppt. In the first function, the means of the number of broods per female (−1.370) at 50 ppt, lifespan (−4.186) at 100 ppt, and lifespan (−2.743) had the main impact on the classification of groups at 150 ppt (Table S4). As illustrated in Figure 3, the tetraploid lineage at 50 ppt, the diploid and tetraploid lineages at 100 ppt, and the diploid lineage at 150 ppt were distinctly separated from the other groups. All the individuals within these groups were 100% correctly classified into their respective lineages. In contrast, the triploid lineage presented the lowest percentage of correctly classified groups at all tested salinities. The highest and lowest percentages of the overall classified groups were observed for the 150 ppt (91.3%) and 100 ppt (86.4%) treatments, respectively (Table 5).

Figure 2B and Table S3 show the differences in reproductive modes across different salinities for each ploidy level. Consistent with the within-lineage findings, we observed no significant association between reproductive mode and ploidy level. The diploid and tetraploid lineages exhibited entirely oviparous reproduction modes at salinities of 50 ppt and 100 ppt, respectively. In contrast, all the treatments at 150 ppt resulted in both oviparity and ovoviviparity, with oviparity being the dominant mode (73.96%) in the tetraploid lineage.

### 3.3. Mixed Groups

**Figure 4:**
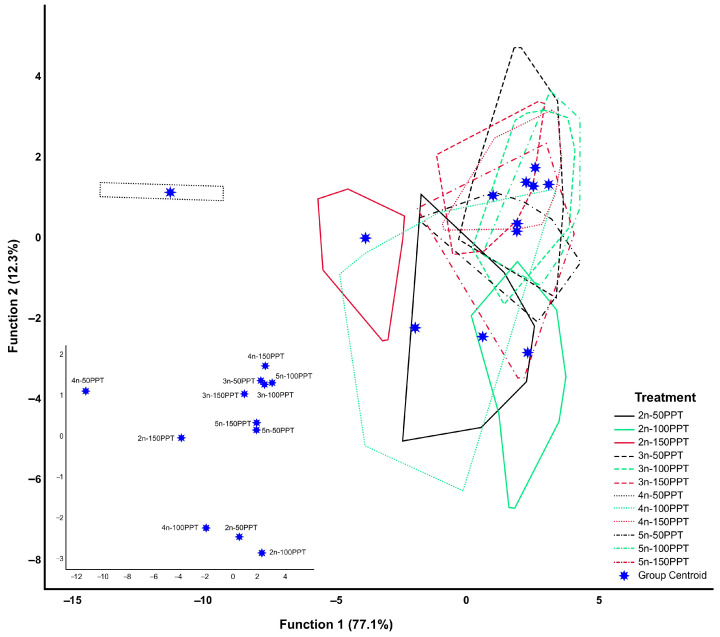
Scatterplot of the first two canonical discriminant functions resulting from mixed groups (among ploidy levels and salinities) from the discriminant analysis (the individuals corresponding to each polygon are presented in Figure S3).

Figure 4 presents the discriminant analysis scatterplot comparing ploidy levels across varying salinity conditions. The results demonstrate that the first discriminant function accounted for 77.1% of the observed variation, and the cumulative contribution of the first and second functions reached 89.4%. The first discriminant function was most strongly influenced by the mean lifespan (−1.883), which drove the separation of groups (Table S5). At 50 ppt, the tetraploid lineage presented an isolated pattern, whereas eight groups, including the triploid and pentaploid lineages, along with the tetraploid lineage at 100 and 150 ppt, clustered together. Partial distribution overlaps were observed between the diploid and tetraploid lineages at 150 ppt and 100 ppt, the diploid and tetraploid lineages at 50 ppt and 100 ppt, and the diploid lineage at 50 ppt and 100 ppt (Figure 4). At 50 ppt, the tetraploid lineage individuals were 100% correctly classified into their respective groups. In contrast, lower classification was observed in the following groups: the pentaploid lineage at 100 ppt (22.9%), the triploid lineage at 100 ppt (34.3%) and 50 ppt (46.7%), and the diploid lineage at 50 ppt (91.4%). Overall, the original classification across groups achieved an accuracy of 68.5% (Table 6).

## 4. Discussion

### 4.1. Reproductive Traits

Browne [33] compared the reproductive traits of two parthenogenetic forms from India (Madras and Kutch) at 60 ppt salinity and 26 °C. The reproductive traits of the two studied *Artemia* parthenogenetic lineages significantly differed, except for “days between broods”. In a later study, Browne et al. [19] broadened the scope to analyze reproductive traits across Old World (*Artemia salina*) and New World (*A. franciscana* and *A. persimilis*) species, which involved seven geographically distinct populations, as well as parthenogenetic *Artemia* from five localities in Asia and Europe, under conditions of 90 ppt salinity and 24 °C. The findings revealed significant differences among the three groups (Old World and New World species and parthenogenetic inhabitants), with pronounced differences observed within the parthenogenetic *Artemia* forms. However, there is insufficient information on the ploidy levels of the studied parthenogenetic groups.

Salinity acts as a critical environmental driver of reproductive traits in *Artemia*. Several studies have demonstrated that salinity changes within relatively high ranges can reduce reproductive potential [21,49,50,51,52] and shorten both the reproductive period and the post-reproductive period [21,25]. Additionally, maturation is delayed due to a prolonged pre-reproductive period under conditions of high salinity [21,25,50]. Triantaphyllidis et al. [21] identified an optimal salinity range of 60–100 ppt for parthenogenetic *Artemia* from the Tanggu salterns. Deviations from this range—whether below or above optimal levels—may similarly impair reproductive potential and delay maturation.

Browne & Wanigasekera [24] studied the combined effects of salinity (60, 120, and 180 ppt) and temperature (15, 24, and 30 °C) on the reproductive performance of parthenogenetic *Artemia* from Margherita di Savoia, Italy. Their results revealed 24 °C and 120 ppt as the optimal conditions for maximizing total offspring. However, that study had a critical limitation, as the studied habitat included a mix of diploid and tetraploid lineages (for more information, see [24]), and the ploidy levels of individuals were not considered; thus, ploidy differences could drive variability in salinity/temperature tolerance or reproductive strategies, complicating efforts to generalize conclusions on adaptive responses.

Abatzopoulos et al. [25] studied a cloned tetraploid parthenogenetic *Artemia* lineage from M. Embolon, Greece. Their results indicated that increasing salinity levels (from 50 to 120 ppt at 22 °C) prolonged the pre-reproductive period while shortening the post-reproductive period. This pattern aligns with findings in other *Artemia* species, such as *A. monica* [49] and *A. urmiana* [27], as well as parthenogenetic *Artemia* from Bohai Bay, China [50,52], and Urmia Lake, Iran [27].

In our study, elevated salinity prolonged the pre-reproductive period in diploid and triploid parthenogenetic lineages. In contrast, pentaploid *Artemia* exhibited extended pre-reproductive periods at both low (50 ppt) and high (150 ppt) salinities. Surprisingly, none of the 35 tetraploid parthenogenetic individuals (average lifespan: 89.80 days) reached reproduction at 50 ppt salinity. A similar finding was observed for parthenogenetic *Artemia* from the lagoons surrounding Urmia Lake (lifespan: 48.3 days), but reproduction was completely inhibited at 175 ppt salinity and 27 °C. This result occurred while Urmia Lake’s resident parthenogenetic lineage produced 2.8 broods per female under 150 ppt salinity, despite having a similar lifespan (42.20), although their ploidy levels were not analyzed (see [27]). Yang & Sun [32] reported that female *A. sinica* failed to reproduce under high salinity (150 and 200 ppt) at 30 °C. However, the absence of lifespan data for these treatments complicates comparisons of reproductive performance across conditions. These findings collectively suggest that critical salinity thresholds, whether low or high, combined with thermal stress, can selectively inhibit reproduction in *Artemia* populations/lineages without inducing early mortality. This phenomenon appears to be context-dependent rather than a common physiological response.

The present study revealed distinct reproductive performance (total offspring) across ploidy levels under varying salinities. The diploid and triploid lineages presented the highest total offspring production at 100 ppt (118.43 offspring) and at 50 and 100 ppt (183.70 offspring and 226.23 offspring, respectively, with a non-significant difference). At 150 ppt, tetraploid *Artemia* presented the highest total number of offspring (315.77 offspring), with the highest value across all tested salinities and ploidy levels. In contrast, compared with the other lineages, the pentaploid lineage produced significantly fewer offspring. However, no significant difference was detected between the triploid and pentaploid lineages at 100 ppt (226.23 vs. 205.11 offspring). Our findings indicated that salinity alterations did not affect the reproductive performance of parthenogenetic lineages identically. In contrast to the known role of ploidy level, particularly compared with the pentaploid lineage, in shaping reproductive strategies, our results suggest that each lineage (ploidy level) exhibits specific adaptations to salinity. However, further studies are needed to clarify the role(s) of ecological and geographical isolation among residents of different habitats within each ploidy level.

### 4.2. Lifespan

While earlier studies have linked lifespan to distinct life phases (pre-reproductive, reproductive, and post-reproductive periods; see Materials and Methods), our results demonstrate that lifespan is independent of these phases. For example, in the diploid lineage, the longest pre-reproductive period was observed at 150 ppt (49.16 days), whereas the maximum lifespan occurred at 100 ppt (83.03 days). Furthermore, the reproductive period at 150 ppt (7.39 days) was significantly shorter than that at 50 ppt (18.31 days), despite a longer lifespan at 150 ppt (67.00 days) than at 50 ppt (58.26 days). A previous study found no significant lifespan difference among parthenogenetic *Artemia* from Qarun Lake, Borg El-Arab, and El-Max at 150 ppt. However, the reproductive period of Qarun Lake *Artemia* was significantly longer than that of *Artemia* from the other lakes [26].

Abatzopoulos et al. [25] examined a cloned tetraploid parthenogenetic *Artemia* lineage (M. Embolon, Greece) across different salinities (50, 80, and 120 ppt) at 22 °C. They reported the highest lifespans at 120 ppt (70.43 days) and 50 ppt (64.43 days), with no statistically significant difference between these two salinities. Baxevanis et al. [26] studied three African parthenogenetic *Artemia* populations (ploidy level was undetermined) and reported the longest lifespan at an intermediate salinity of 120 ppt (ranging from 35 ppt to 200 ppt).

Our results revealed no significant correlation between salinity level and lifespan across ploidy levels. However, the diploid and pentaploid lineages achieved their maximum lifespan at 100 ppt. The triploid lineage displayed similar peak lifespans at both 100 ppt and 150 ppt, whereas the tetraploid lineage achieved a maximum lifespan at 150 ppt. Notably, no consistent relationship emerged between ploidy level and lifespan across the studied groups. The maximum lifespan values at 50, 100, and 150 ppt were recorded in the tetraploid, di-/tri-/tetraploid, and tetraploid lineages, respectively. In unambiguous contrast, the pentaploid lineage consistently presented the shortest lifespans across all salinities.

### 4.3. Reproductive Mode

Gajardo & Beardmore [20] demonstrated that oviparity in *A. franciscana* from Great Salt Lake (USA) was positively correlated with female heterozygosity levels. Subsequent studies on parthenogenetic *Artemia*, including those from Hoh Salt Lake, China [53], Caka Salt Lake, China [54], and *A. salina* from Larnaca Salt Lake, Cyprus [24], suggested that oviparity may occur naturally in certain wild individuals. Moreover, extensive laboratory studies highlight the critical role of physicochemical parameters (e.g., salinity, temperature, photoperiod, and dissolved oxygen) and food availability in modulating reproductive modes (oviparity vs. ovoviviparity) in both sexual *Artemia* species and parthenogenetic lineages (see [30,32,55]).

Berthélémy-Okazaki & Hedgecock [56] reported that oviparity in *A. franciscana* (San Francisco Bay, USA) decreases with increasing salinity following a combination of temperatures. A diploid clone of parthenogenetic *Artemia* from Barkol Salt Lake (China) demonstrated that at 25 °C and a short light period (6 h), the percentage of oviparous individuals was 83.33% at 70 ppt and 93.86% at 140 ppt [57]. In parthenogenetic *Artemia* from Putian saltern (Fujian, China) and Ebinur Lake (also known as AiBi Lake, Xinjiang, China), increasing salinity initially led to higher oviparity rates, followed by subsequent population declines [53]. In contrast, elevated salinity was correlated with sustained increases in oviparity rates in populations from Greece [25] and Bohai Bay, China [52]. However, only the ploidy level of parthenogenetic *Artemia* from Greece, a triploid lineage, was determined.

Yang & Sun [32] reported that salinity, as well as the interactions of salinity/temperature and salinity/photoperiod, influenced the percentage of oviparous offspring in *A. sinica* (Yuncheng Salt Lake, China), which, under low temperature conditions (16 °C), a short photoperiod (6 h), and low salinity (50 ppt and 100 ppt), dominated oviparous reproduction (87.9–85.7%). However, *A. sinica* did not exhibit fully oviparous or ovoviviparous reproduction under any of the examined conditions. In contrast, Jia et al. [51] reported that at salinities above 100 ppt, the reproductive mode of *A. sinica* (Yuncheng Salt Lake, China) changed to oviparity. We hypothesize that the contradictory results observed among populations of a species, or even within a single population of a species, may arise from interspecific variation and/or the effect of environmental/physicochemical conditions on the quality of *Artemia* eggs produced. Furthermore, the sampling date and storage conditions of *Artemia* eggs may influence embryo quality and, ultimately, reproductive performance.

Drinkwater & Clegg [58] proposed that ovoviviparity in *Artemia* acts as an adaptive strategy to maintain generational continuity under extreme environmental conditions because their eggs cannot hatch at high salinities (≥120 ppt), resulting in ovoviviparity—the live birth of fully developed larvae—a mechanism to bypass this limitation within the same season. Furthermore, beyond this hypothesized adaptation, seasonal precipitation may play a supplementary role. Rainfall can induce temporary reductions in salinity within the upper water layer, creating localized conditions suitable for egg hatching. This interaction between reproductive strategy and environmental variability warrants consideration in explaining *Artemia* population dynamics (Atashbar, personal observation).

Although oviparity has been proposed as an adaptive strategy to stabilize reproduction under high salinity and ensure the survival of the next generation (for more information, see [59,60,61]), our findings indicate that there is no relationship between salinity or ploidy level and the rate of oviparity. The diploid and tetraploid lineages exhibited fully oviparous reproduction at 50 ppt and 100 ppt, whereas the triploid and pentaploid lineages presented markedly lower oviparity rates (<50%) under the same conditions. Furthermore, the reproductive mode remained independent of ploidy level at identical salinities.

While *A. sinica* exhibited oviparity under conditions of abundant food availability [62], Zhang et al. [63] reported contrasting findings for the same species. Vahdat [55] reported that dietary supplementation with vinasse (a byproduct of sugar production) slightly stimulated oviparity in a parthenogenetic lineage (unknown ploidy level), although its effect was significantly more pronounced in *A. franciscana*. Our results emphasize that the reproductive mode of parthenogenetic *Artemia* cannot be predicted solely by salinity or ploidy level. Instead, it might be shaped by dynamic interactions between genetic lineages, the ecological context, and physicochemical stressors. Future research should prioritize longitudinal field studies across geographically isolated habitats, coupled with controlled experiments addressing multifactorial stressors (e.g., salinity, temperature, and photoperiod interactions), to disentangle the mechanisms driving changes in reproductive strategy in these ecologically vital crustaceans.

## 5. Conclusions

In contrast to Browne [33] and Zhang & King [64], who suggested that the ploidy level of parthenogenetic *Artemia* is linked to tolerance under critical conditions (e.g., salinity), our results, which examined all available ploidy levels, demonstrated no positive correlation between salinity tolerance and ploidy level. Specifically, the pentaploid lineage presented the shortest lifespan across all salinities. The demographic structures of the triploid and pentaploid lineages under varying salinities presented partially overlapping patterns within their respective groups, whereas the diploid and tetraploid lineages presented predominantly scattered distributions. It can be concluded that the life history dynamics of parthenogenetic *Artemia* are shaped by a complex interplay of ploidy level, salinity, nutritional/food resources, and environmental stressors, with no correlated pattern applicable across only the ploidy level of lineages. As prior studies have not systematically compared reproductive and lifespan traits across different ploidy levels, we highlight critical open questions: Are the observed differences intrinsic to specific ploidy lineages, or could they shift among geographically isolated habitats occupied by lineages of the same ploidy level? Furthermore, might temporal sampling within the same habitat reveal dynamic changes in these traits? Our findings underscore the need for broader comparative studies to disentangle the roles of ploidy level, geography, temporal adaptation, and environmental variation in shaping *Artemia* resilience.

## Figures and Tables

**Table 1:** List of studied parthenogenetic *Artemia* lineages, their provenance, and egg bank accession. (1) Ocean University of China, (2) *Artemia* Reference Center.

Ploidy Level	Locality	Geographic Coordinates	Abb.	Egg Bank Accession
Diploid lineage	Ga Hai Lake, China	37°08′ N, 97°33′ E	2n	OUC ^1^, China
Triploid lineage	Ankiembe Saltern, Madagascar	23°23′ S, 43°42′ E	3n	ARC ^2^, Belgium
Tetraploid lineage	Hoh Lake, China	36°56′ N, 98°12′ E	4n	OUC, China
Pentaploid lineage	Yinggehai Saltern, China	18°31′ N, 108°44′ E	5n	OUC, China

**Table 2:** Mean (SD) values of reproductive and lifespan traits for the parthenogenetic lineages at three salinities. (A) pre-reproductive period, (B) reproductive period, (C) post-reproductive period, (D) number of broods per female, (E) interval between broods, (F) number of nauplii, (G) number of eggs, (H) total offspring, (I) offspring per brood, and (J) lifespan. Within each ploidy group, the same letter indicates no significant differences at *p* > 0.05.

	2n			3n			4n			5n		
Trait	50 ppt n = 35	100 ppt n = 35	150 ppt n = 31	50 ppt n = 30	100 ppt n = 35	150 ppt n = 32	50 ppt n = 35	100 ppt n = 35	150 ppt n = 35	50 ppt n = 28	100 ppt n = 35	150 ppt n = 28
**A**	26.29 ^a^ (6.11)	21.14 ^b^ (3.73)	49.16 ^c^ (13.73)	21.77 ^a^ (4.47)	22.31 ^a^ (3.11)	30.94 ^b^ (4.45)	88.80 ^a^ (9.41)	45.57 ^b^ (8.12)	23.54 ^c^ (5.63)	24.11 ^b^ (3.83)	19.29 ^a^ (2.31)	22.79 ^b^ (3.32)
**B**	18.31 ^a^ (10.62)	46.51 ^b^ (9.06)	7.39 ^c^ (8.79)	37.60 ^a^ (27.54)	54.51 ^b^ (28.00)	47.81 ^ab^ (19.55)	0.00 ^a^ (0.00)	15.57 ^b^ (10.94)	65.23 ^c^ (13.51)	12.54 ^a^ (13.84)	39.77 ^b^ (17.91)	21.82 ^a^ (17.35)
**C**	13.66 ^b^ (8.91)	15.37 ^b^ (8.11)	6.29 ^a^ (5.16)	2.03 ^a^ (4.00)	4.91 ^a^ (5.55)	4.13 ^a^ (4.80)	0.00 ^a^ (0.00)	21.51 ^b^ (14.97)	9.51 ^c^ (5.42)	5.96 ^a^ (6.30)	7.91 ^a^ (9.46)	5.00 ^a^ (9.49)
**D**	2.94 ^a^ (1.14)	5.37 ^b^ (0.77)	2.19 ^c^ (1.42)	7.83 ^a^ (5.43)	10.69 ^b^ (4.98)	9.31 ^ab^ (3.45)	0.00 ^a^ (0.00)	2.94 ^b^ (0.97)	13.17 ^c^ (2.35)	3.18 ^a^ (2.64)	9.31 ^b^ (4.00)	5.11 ^a^ (3.42)
**E**	8.07 ^a^ (4.07)	10.06 ^b^ (2.29)	2.26 ^c^ (2.71)	3.91 ^a^ (2.15)	4.44 ^a^ (0.96)	4.61 ^a^ (0.84)	0.00 ^a^ (0.00)	6.03 ^b^ (3.79)	4.27 ^c^ (0.62)	2.83 ^a^ (2.30)	3.50 ^a^ (1.08)	3.47 ^a^ (1.63)
**F**	0.00 ^a^ (0.00)	9.89 ^ab^ (33.34)	14.35 ^b^ (13.28)	160.90 ^a^ (126.62)	114.83 ^a^ (79.34)	105.41 ^a^ (70.41)	0.00 ^a^ (0.00)	0.00 ^a^ (0.00)	82.80 ^b^ (39.51)	39.96 ^a^ (44.38)	132.51 ^b^ (109.71)	55.86 ^a^ (59.44)
**G**	30.60 ^a^ (13.69)	108.54 ^b^ (42.68)	2.13 ^c^ (6.26)	22.80 ^a^ (45.28)	111.40 ^b^ (108.69)	93.34 ^b^ (87.05)	0.00 ^a^ (0.00)	46.14 ^b^ (21.91)	232.97 ^c^ (58.30)	30.00 ^a^ (64.26)	72.60 ^a^ (114.27)	36.79 ^a^ (51.80)
**H**	30.60 ^a^ (13.69)	118.43 ^b^ (26.90)	16.48 ^c^ (13.48)	183.70 ^a^ (138.04)	226.23 ^a^ (111.20)	198.75 ^a^ (87.50)	0.00 ^a^ (0.00)	46.14 ^b^ (21.91)	315.77 ^c^ (59.72)	69.96 ^a^ (66.83)	205.11 ^b^ (98.59)	92.64 ^a^ (68.83)
**I**	10.48 ^a^ (2.97)	22.10 ^b^ (4.26)	6.97 ^c^ (4.02)	21.84 ^a^ (5.88)	21.09 ^a^ (4.69)	20.64 ^a^ (4.12)	0.00 ^a^ (0.00)	15.37 ^b^ (3.16)	24.14 ^c^ (3.41)	20.63 ^ab^ (8.29)	21.47 ^b^ (5.33)	17.07 ^a^ (5.56)
**J**	58.26 ^a^ (11.40)	83.03 ^b^ (9.02)	67.00 ^c^ (10.29)	64.63 ^a^ (33.47)	82.66 ^b^ (30.64)	84.09 ^b^ (22.88)	89.80 ^a^ (9.41)	82.66 ^a^ (16.05)	98.29 ^b^ (17.17)	43.21 ^a^ (18.35)	67.69 ^b^ (21.52)	50.46 ^a^ (21.95)

**Table 3:** Classification results of complete original individuals for each ploidy level at different salinities (within groups) by discriminant analysis.

Ploidy Level	Category	Treatment	50 ppt	100 ppt	150 ppt	Total
**2n ^a^**	Count	50 ppt	**35**	0	0	35
		100 ppt	1	**34**	0	35
		150 ppt	0	0	**31**	31
	%	50 ppt	**100**	0	0	100
		100 ppt	2.9	**97.1**	0	100
		150 ppt	0	0	**100**	100
**3n ^b^**	Count	50 ppt	**25**	3	2	30
		100 ppt	7	**25**	3	35
		150 ppt	1	2	**29**	32
	%	50 ppt	**83.3**	10	6.7	100
		100 ppt	20	**71.4**	8.6	100
		150 ppt	3.1	6.3	**90.6**	100
**4n ^c^**	Count	50 ppt	**35**	0	0	35
		100 ppt	0	**35**	0	35
		150 ppt	0	0	**35**	35
	%	50 ppt	**100**	0	0	100
		100 ppt	0	**100**	0	100
		150 ppt	0	0	**100**	100
**5n ^d^**	Count	50 ppt	**16**	1	11	28
		100 ppt	2	**28**	5	35
		150 ppt	6	4	**18**	28
	%	50 ppt	**57.1**	3.6	39.3	100
		100 ppt	5.7	**80**	14.3	100
		150 ppt	21.4	14.3	**64.3**	100

(a) In total, 99.0% of the original grouped cases were correctly classified. (b) In total, 81.4% of the original grouped cases were correctly classified. (c) In total, 100.0% of the original grouped cases were correctly classified. (d) In total, 68.1% of the original grouped cases were correctly classified.

**Table 4:** The statistical analyses of reproductive and lifespan traits among different ploidy levels at the same salinity. Within each salinity group, the same letter indicates no significant differences at *p* > 0.05. Abbreviations are listed in Table 2.

	50 ppt				100 ppt				150 ppt			
Trait	2n n = 35	3n n = 30	4n n = 35	5n n = 28	2n n = 35	3n n = 35	4n n = 35	5n n = 35	2n n = 31	3n n = 32	4n n = 35	5n n = 28
**A**	b	a	c	ab	a	a	b	a	c	b	a	a
**B**	b	c	a	b	bc	c	a	b	a	b	c	d
**C**	c	a	a	b	b	a	b	a	ab	a	b	a
**D**	b	c	a	b	b	c	a	c	a	b	c	d
**E**	c	b	a	b	c	a	b	a	a	c	bc	b
**F**	a	b	a	a	a	b	a	b	a	c	bc	b
**G**	b	ab	a	b	b	b	a	ab	a	b	c	a
**H**	ab	c	a	b	b	c	a	c	a	b	c	d
**I**	b	c	a	c	b	b	a	b	a	b	c	d
**J**	b	b	c	a	b	b	b	a	a	b	c	d

**Table 5:** Classification results of complete original individuals of each salinity at different ploidy levels (between groups) by discriminant analysis.

Treatment	Category	Ploidy Level	2n	3n	4n	5n	Total
**50 ppt ^a^**	Count	2n	**33**	0	0	2	35
		3n	0	**18**	0	12	30
		4n	0	0	**35**	0	35
		5n	1	1	0	**26**	28
	%	2n	**94.3**	0	0	5.7	100
		3n	0	**60**	0	40	100
		4n	0	0	**100**	0	100
		5n	3.6	3.6	0	**92.9**	100
**100 ppt ^b^**	Count	2n	**35**	0	0	0	35
		3n	0	**23**	0	12	35
		4n	0	0	**35**	0	35
		5n	1	6	0	**28**	35
	%	2n	**100**	0	0	0	100
		3n	0	**65.7**	0	34.3	100
		4n	0	0	**100**	0	100
		5n	2.9	17.1	0	**80**	100
**150 ppt ^c^**	Count	2n	**31**	0	0	0	31
		3n	0	**24**	3	5	32
		4n	0	1	**34**	0	35
		5n	0	1	1	**26**	28
	%	2n	**100**	0	0	0	100
		3n	0	**75**	9.4	15.6	100
		4n	0	2.9	**97.1**	0	100
		5n	0	3.6	3.6	**92.9**	100

(a) In total, 87.5% of the original grouped cases were correctly classified. (b) In total, 86.4% of the original grouped cases were correctly classified. (c) In total, 91.3% of the original grouped cases were correctly classified.

**Table 6:** Classification results of complete original individuals of each ploidy level at each salinity (mixed groups) by discriminant analysis.

			2n			3n			4n			5n			
Category	Ploidy Level	Treatment	50 ppt	100 ppt	150 ppt	50 ppt	100 ppt	150 ppt	50 ppt	100 ppt	150 ppt	50 ppt	100 ppt	150 ppt	Total
**Count**	**2n**	**50 ppt**	**32**	0	1	0	0	0	0	1	0	1	0	0	35
		**100 ppt**	1	**33**	0	0	0	0	0	0	0	1	0	0	35
		**150 ppt**	0	0	**29**	0	0	0	0	2	0	0	0	0	31
	**3n**	**50 ppt**	0	0	0	**14**	0	3	0	0	1	7	1	4	30
		**100 ppt**	1	0	0	3	**12**	0	0	0	10	5	2	2	35
		**150 ppt**	0	0	0	1	2	**20**	0	0	3	1	0	5	32
	**4n**	**50 ppt**	0	0	0	0	0	0	**35**	0	0	0	0	0	35
		**100 ppt**	3	1	2	0	0	0	0	**27**	0	1	0	1	35
		**150 ppt**	0	0	0	0	4	1	0	0	**29**	0	1	0	35
	**5n**	**50 ppt**	0	1	1	0	1	0	0	0	1	**17**	0	7	28
		**100 ppt**	0	0	0	10	3	0	0	0	7	3	**8**	4	35
		**150 ppt**	1	0	1	2	3	0	0	0	1	3	3	**14**	28
**%**	**2n**	**50 ppt**	**91.4**	0	2.9	0	0	0	0	2.9	0	2.9	0	0	100
		**100 ppt**	2.9	**94.3**	0	0	0	0	0	.0	0	2.9	0	0	100
		**150 ppt**	0	0	**93.5**	0	0	0	0	6.5	0	.0	0	0	100
	**3n**	**50 ppt**	0	0	0	**46.7**	0	10	0	0	3.3	23.3	3.3	13.3	100
		**100 ppt**	2.9	0	0	8.6	**34.3**	0	0	0	28.6	14.3	5.7	5.7	100
		**150 ppt**	0	0	0	3.1	6.3	**62.5**	0	0	9.4	3.1	0	15.6	100
	**4n**	**50 ppt**	0	0	0	0	0	0	**100**	0	0	0	0	0	100
		**100 ppt**	8.6	2.9	5.7	0	0	0	0	**77.1**	0	2.9	0	2.9	100
		**150 ppt**	0	0	0	0	11.4	2.9	0	0	**82.9**	0	2.9	0	100
	**5n**	**50 ppt**	0	3.6	3.6	0	3.6	0	0	0	3.6	**60.7**	0	25	100
		**100 ppt**	0	0	0	28.6	8.6	0	0	0	20	8.6	**22.9**	11.4	100
		**150 ppt**	3.6	0	3.6	7.1	10.7	0	0	0	3.6	10.7	10.7	**50**	100

In total, 68.5% of the original grouped cases were correctly classified.

## Data Availability

The original contributions presented in this case are included in the article. Further inquiries can be directed to the corresponding authors.

## Supplementary Materials

The following supporting information can be downloaded at: https://www.mdpi.com/article/10.3390/biology14081055/s1, Table S1: maximum/minimum values of reproductive and lifespan traits for the parthenogenetic lineages at three salinities. Abbreviations are listed in Table 2; Table S2: Standardized canonical discriminant function coefficients of each ploidy level at different salinities (within groups). Abbreviations are listed in Table 2; Table S3: Percentages of oviparous offspring (SSD) of females with different ploidy levels at different salinities. The same lowercase letters indicate non-significant differences in each row among different salinities at the same ploidy level (*p* > 0.05). The same uppercase letter indicates non-significant differences in each row among different ploidy levels at the same salinity (*p* > 0.05). Abbreviations are listed in Table 2; Table S4: Standardized canonical discriminant function coefficients of each salinity at different ploidy levels (between groups). Abbreviations are listed in Table 2; Table S5: Standardized canonical discriminant function coefficients of each salinity and ploidy level (mixed groups). Abbreviations are listed in Table 2. Figure S1: Scatterplot (with different salinities at the same ploidy level) from the discriminant analysis based on individuals corresponding to each polygon (treatment). Abbreviations are available in Figure 1. Figure S2: Scatterplot (among ploidy levels at the same salinity) from the discriminant analysis based on individuals corresponding to each polygon (treatment). Abbreviations are available in Figure 3. Figure S3: Scatterplot (among ploidy levels and salinities) from the discriminant analysis based on individuals corresponding to each polygon (treatment). Abbreviations are available in Figure 4.
