# Supplementary material for: Effects of Salinity on the Reproductive and Lifespan Traits of Artemia Parthenogenetic Lineages with Different Ploidy Levels
Source: Biology (Basel). 2025 Aug 15;14(8):1055. doi: 10.3390/biology14081055 (PMC12383873; doi:10.3390/biology14081055)
Supplement: Supplementary file 1 [file biology-14-01055-s001.zip › biology-3735890-supplementary.pdf]

Table S1. maximum/minimum values of reproductive and lifespan traits for the parthenogenetic lineages at three salinities. Abbreviations are listed in Table 2.

	2n			3n			4n			5n		
**Trait**	50 ppt n = 35	100 ppt n = 35	150 ppt n = 31	50 ppt n = 30	100 ppt n = 35	150 ppt n = 32	50 ppt n = 35	100 ppt n = 35	150 ppt n = 35	50 ppt n = 28	100 ppt n = 35	150 ppt n = 28
**A**	17/43	15/32	0/62	17/38	15/28	24/45	78/104	32/61	20/43	19/35	16/26	17/32
**B**	1/40	22/62	0/39	1/87	5/88	4/77	0/0	4/42	35/81	1/52	1/63	1/51
**C**	0/38	0/38	0/20	0/17	0/26	0/16	0/0	1/51	0/21	0/28	0/39	0/47
**D**	1/5	4/7	0/6	1/16	2/17	2/17	0/0	2/5	9/16	1/12	1/16	1/12
**E**	0/17	4.25/15.5	0/9	0/8	2/7.5	2/6.8	0/0	2/15.5	3.25/6.1	0/9	0/5.83	0/7
**F**	0/0	0/141	0/51	6/431	12/328	0/255	0/0	0/0	16/175	0/186	0/341	0/195
**G**	6/65	0/184	0/32	0/213	0/335	0/312	0/0	19/122	114/348	0/292	0/363	0/192
**H**	6/65	60/184	0/52	6/451	34/388	18/340	0/0	19/122	175/424	1/317	11/363	2/233
**I**	5.33/17	14.71/32.4	0/14	6/29.62	10.36/28.27	9/31.2	0/0	9.5/24.4	15.91/31.64	1/32.33	7.1/30.56	2/33.5
**J**	38/80	60/95	52/101	20/120	25/123	30/115	79/105	40/103	57/113	26/99	23/99	23/99

Table S2. Standardized canonical discriminant function coefficients of each ploidy level at different salinities (within groups). Abbreviations are listed in Table 2.

	2n		3n		4n		5n	
Trait	Function1	Function2	Function1	Function2	Function1	Function2	Function1	Function2
A	-0.283	0.256	1.119	0.227	-0.272	-0.030	0.812	0.494
B	1.386	-0.343	0.536	2.694	-1.161	0.769	0.243	3.069
C	0.960	-0.683	0.204	1.061	0.245	-0.519	0.274	2.044
D	-0.236	-0.638	1.563	3.258	2.601	-0.758	-0.077	-0.130
E	0.062	-0.388	0.063	0.373	0.733	-0.809	0.527	-0.719
F	0.370	1.129	-1.209	-1.721	-0.996	0.815	-0.685	0.213
G	0.456	1.423	-0.763	-0.731	-1.690	1.181	-0.434	0.048
I	0.252	-0.319	-0.020	0.128	1.550	-.0549	0.036	0.890
J	-1.143	1.167	-1.098	-4.707	-0.272	-0.030	-0.613	-4.210

Table S3. Percentages of oviparous offspring (SD) of females with different ploidy levels at different salinities. The same lowercase letters indicate no significant differences in each row among different salinities at the same ploidy level (P > 0.05). The same uppercase letter indicates no significant differences in each row among different ploidy levels at the same salinity (P > 0.05). Abbreviations are listed in Table 2.

2n			3n			4n			5n		
**50 ppt**	**100 ppt**	**150 ppt**	**50 ppt**	**100 ppt**	**150 ppt**	**50 ppt**	**100 ppt**	**150 ppt**	**50 ppt**	**100 ppt**	**150 ppt**
100aA (0.00)	91.42aA (28.40)	13.92bA (30.87)	10.52aB (17.64)	41.92bB (31.50)	43.91bB (30.48)	0.00aB (0.00)	100bA (0.00)	73.96cC (12.16)	42.15aC (48.16)	35.41aB (44.54)	36.08aB (41.68)

Table S4. Standardized canonical discriminant function coefficients of each salinity at different ploidy levels (between groups). Abbreviations are listed in Table 2.

	**50 ppt**		**100 ppt**		**150 ppt**	
**Trait**	Function 1	Function 2	Function 1	Function 2	Function 1	Function 2
**A**	0.678	0.153	1.837	0.024	-0.319	0.235
**B**	-0.226	0.319	4.001	3.231	2.440	-0.871
**C**	-0.178	0.825	2.374	1.017	0.902	-0.586
**D**	-1.370	0.132	-0.237	-1.177	0.147	-0.226
**E**	-0.067	0.780	0.272	0.028	-0.284	0.048
**F**	0.960	-0.576	-0.643	-1.035	-0.080	0.648
**G**	0.596	-0.247	-0.508	-0.936	0.072	1.213
**I**	-0.555	-0.400	0.083	0.515	0.524	-0.402
**J**	0.638	-0.439	-4.186	-1.467	-2.743	1.220

Table S5. Standardized canonical discriminant function coefficients of each salinity and ploidy level (mixed groups). Abbreviations are listed in Table 2.

Trait	**Function 1**	**Function 2**
**A**	-0.417	0.114
**B**	1.187	-0.118
**C**	0.742	-0.493
**D**	1.096	0.359
**E**	0.048	-0.668
**F**	-0.587	0.677
**G**	-0.610	0.543
**I**	0.581	-0.049
**J**	-1.883	-0.273

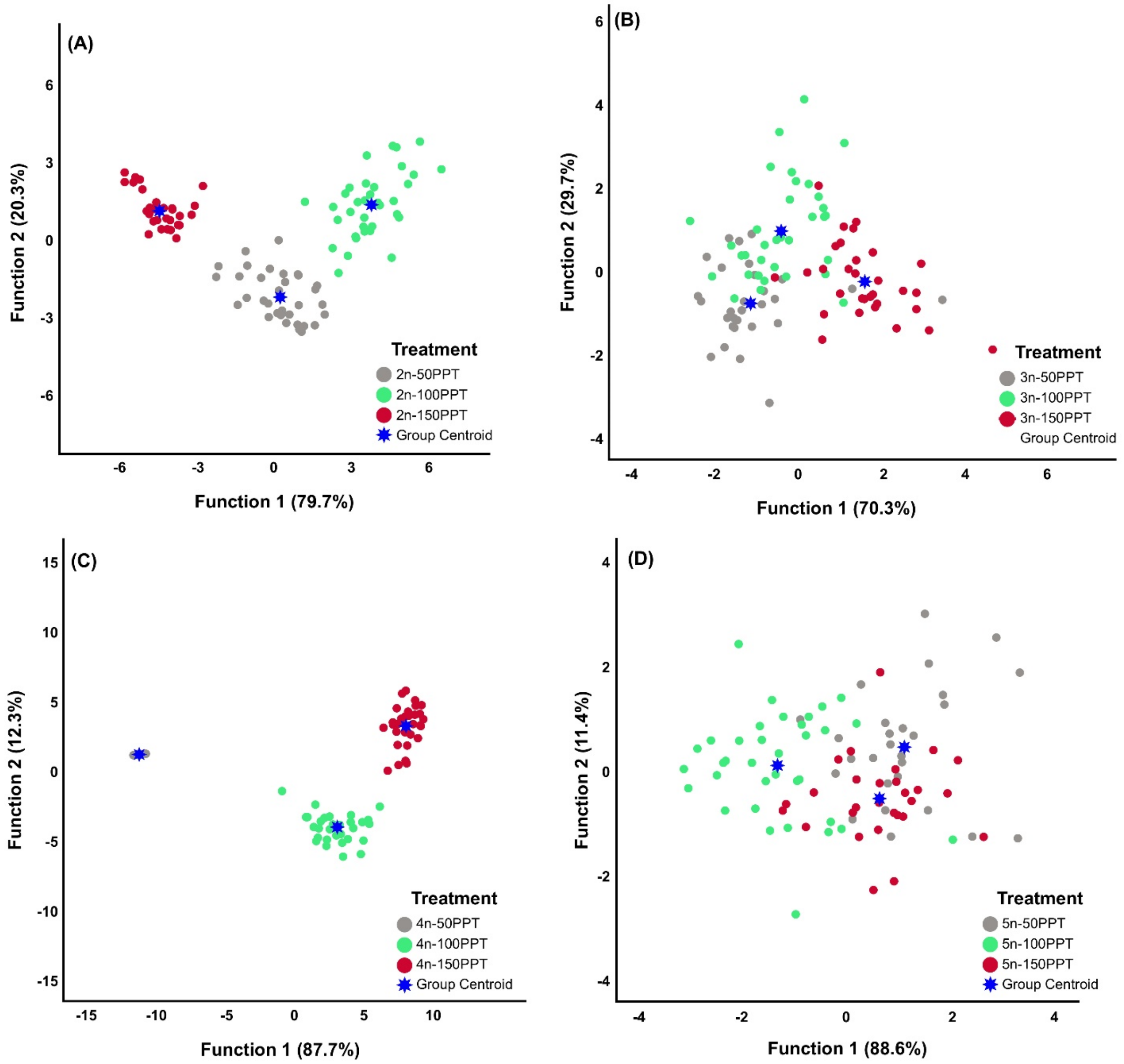

Figure S1: Scatterplot (with different salinities at the same ploidy level) from the discriminant analysis based on individuals corresponding to each polygon (treatment). Abbreviations are available in Figure 1.

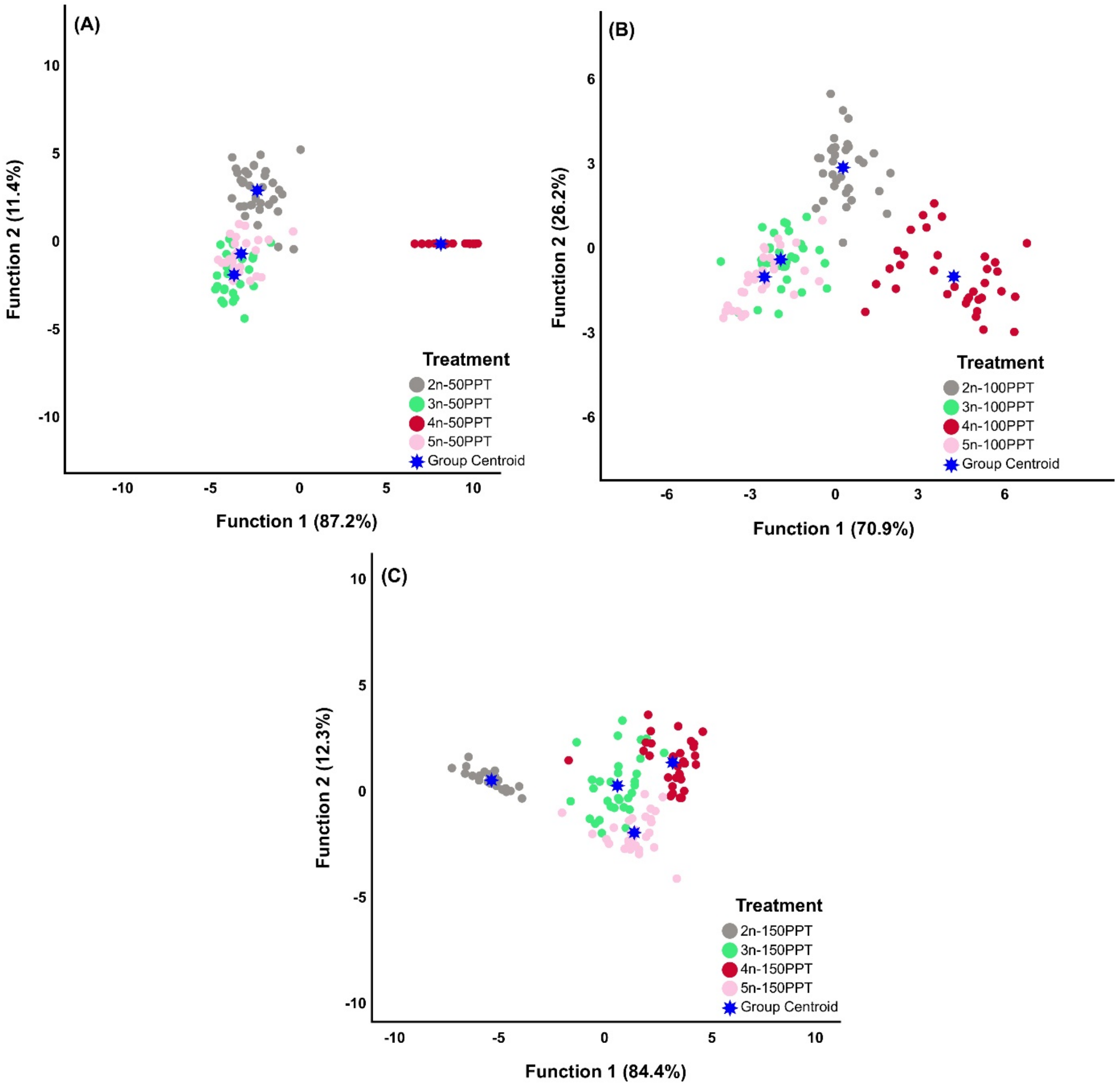

Figure S2: Scatterplot (among ploidy levels at the same salinity) from the discriminant analysis based on individuals corresponding to each polygon (treatment). Abbreviations are available in Figure 3.

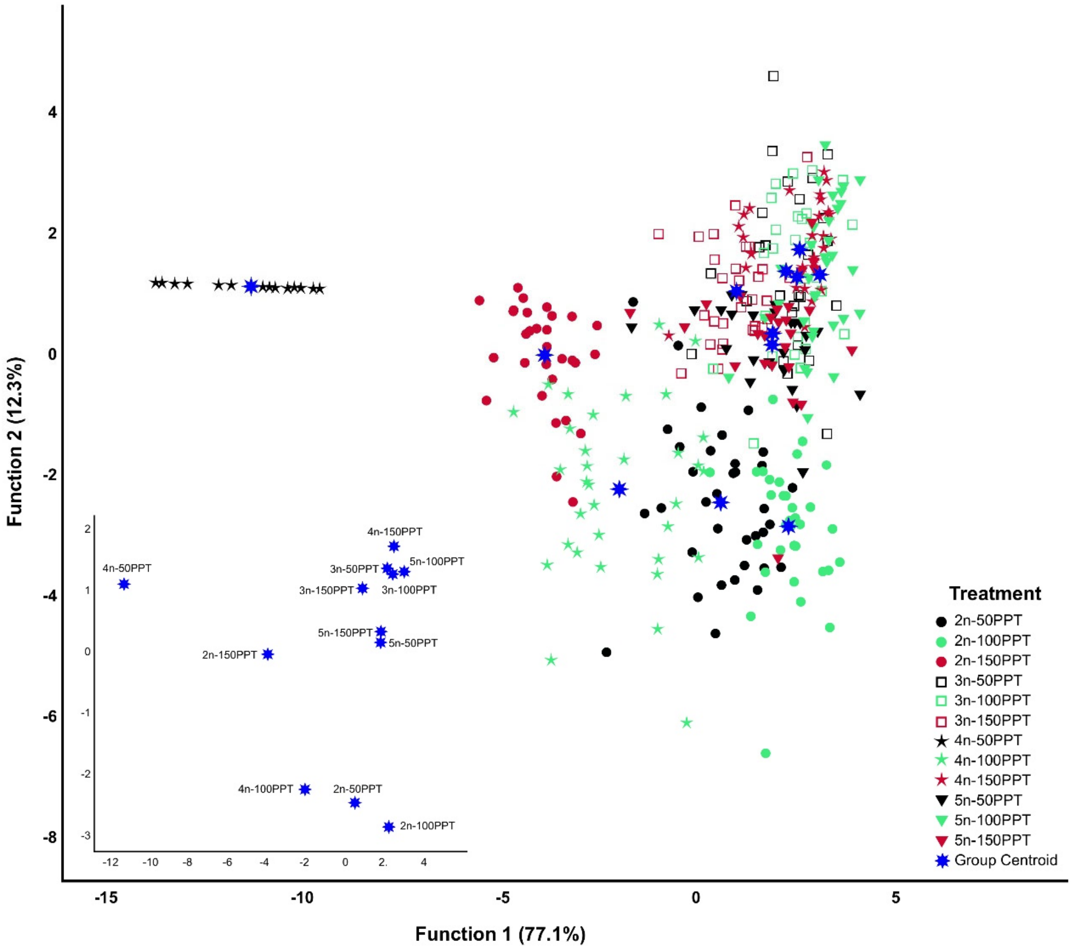

Figure S3: Scatterplot (among ploidy levels and salinities) from the discriminant analysis based on individuals corresponding to each polygon (treatment). Abbreviations are available in Figure 4.
